# The Effects of Diabetic Duration on Lacrimal Functional Unit in Patients with Type II Diabetes

**DOI:** 10.1155/2019/8127515

**Published:** 2019-01-10

**Authors:** Xiaoyu Zeng, Ying Lv, Zhongxiu Gu, Zhe Jia, Chen Zhang, Xiaoxiao Lu, Chenchen Chu, Yichen Gao, Yu Nie, Yanxia Wang, Yan Zhang, Shaozhen Zhao

**Affiliations:** Tianjin Medical University Eye Hospital, Tianjin Medical University Eye Institute, College of Optometry and Ophthalmology, Tianjin Medical University, Tianjin 300384, China

## Abstract

**Purpose:**

To observe ocular surface changes in Type II diabetic patients with different disease durations and to understand the correlations between clinical parameters and diabetic durations.

**Methods:**

In this cross-sectional, prospective study, 51 healthy controls and 91 patients with Type II diabetes were enrolled. The diabetics were divided into 3 subgroups according to the disease duration, including duration <10 y group, 10 to 20 y group, and ≥21 y group. All subjects underwent clinical ocular examinations, including lipid layer thickness (LLT), blinking rate, tear meniscus height (TMH), noninvasive tear film break-up time (NI-BUT), meibography, superficial punctate keratopathy (SPK) scoring, corneal sensitivity, and Schirmer I test. They were also evaluated using the standard patient evaluation of eye dryness (SPEED) questionnaire.

**Results:**

SPEED score, meiboscore, SPK score, LLT, Schirmer I test, and corneal sensitivity differed significantly between the diabetic and healthy control groups. Further, SPEED score, Schirmer I test, corneal sensitivity, meiboscore, and blink rate significantly differed among the 3 diabetic subgroups and the control group. In diabetics, the SPEED score correlated with the SPK score, blink rate, TMH, and LLT; NI-BUT with TMH, LLT, and blink rate; TMH with the SPK score; Schirmer I test with the SPK score; and corneal sensitivity with the meiboscore. More importantly, the Schirmer I test, corneal sensitivity, and SPEED score negatively correlated with diabetic duration.

**Conclusion:**

Diabetic duration is an important factor that affects functions of the lacrimal functional unit in patients with Type II diabetes. The trends of changes in the ocular parameters vary along the course of diabetes.

## 1. Introduction

The lacrimal function unit (LFU) is composed of ocular surface (cornea, conjunctiva, and meibomian glands), lacrimal gland, and a neural network that connects them. It protects lipid, aqueous, and mucin layers of tear film and maintains normal function of ocular surface [1]. If any component of the LFU was damaged, it could lead to reduced tear production, abnormalities in blinking, and changes in tear film composition [2]. The individuals with long-standing hyperglycemia are at an increased risk of developing LFU dysfunction. Clinical studies have demonstrated that diabetic patients were more susceptible to ocular surface disorders than healthy subjects. For instance, keratoepitheliopathy was evident [3–7]; corneal sensitivity [7–11], quantity, and quality of tear secretion [3, 7, 11–14] were reduced in diabetic patients; moreover, the alterations in tear composition [15–18] were also detected in the diabetics. However, the factors contributing to the LFU dysfunction are not clear, and they might include the patient's general condition such as age and gender [5, 19–21], metabolic control [3, 12, 22, 23], duration of diabetes [20, 21], and occurrence of diabetic microvascular complications [5, 8, 22, 23].

The diabetic duration is considered one of the most important risk factors for retinopathy [21, 24, 25], yet its role in the LFU dysfunction remains controversial. Several studies reported no relationship between duration of diabetes and tear functions [3, 12, 13, 22, 23]; whereas others indicated higher prevalence of dry eye syndrome in the patients with longer duration of diabetes [21]; further, a correlation was found between diabetic duration and deterioration of ocular surface clinical parameters [20].

Therefore, this study sought to examine and compare the clinical parameters of ocular surface in the diabetic patients divided into 3 subgroups according to duration of the disease. The correlation between the diabetic duration and clinical ocular surface parameters was also assessed.

## 2. Materials and Methods

### 2.1. Patients

One hundred eight-two eyes of 91 patients with Type II diabetes (diabetic group) and 102 eyes of 51 normal individuals (control group) were enrolled in the current study at Tianjin Medical University Eye Hospital (Tianjin, China) between July and September in 2017. Due to the relatively small number of middle-aged and young people suffering from Type II diabetes and the fact that the duration of diabetes in the middle-aged and young diabetic patients is usually not long enough to reflect the influence of the disease duration on ocular surface functions, the diabetic patients with an average age of 65.43 ± 6.31 y (range 55 to 80 y) were recruited, matching the control group (average age 64.35 ± 5.66 y). Moreover, the diabetic patients should have dry eye symptoms (SPEED ≥ 1), should not resort to ocular medication or surgery within the past 3 m, and should be without ocular injury and diseases including infection, allergy, glaucoma, and autoimmune diseases, and with no history of systemic diseases or administration of systemic medications, such as sex hormone replacement, parasympathomimetics, and parasympatholytics, that may affect tear production or quality. Diabetes was confirmed in all patients by the Department of Internal Medicine; glycosylated hemoglobin levels in these patients were less than 7.8%. The diabetics were further divided into 3 subgroups according to the disease duration: duration <10 y group, duration 10 to 20 y group, and duration ≥21 y group. Retinal status was evaluated by indirect ophthalmoscopy exam and fluorescein angiography, and no PDR was detected in the patients according to the early treatment diabetic retinopathy study criteria [26].

Written informed consent was obtained from all the participants enrolled in this study after a thorough explanation of the study objective and methods. All procedures of this study were approved by the ethical committee in Tianjin Medical University Eye Hospital (Ethical no.: 2017KY (L)-18) and in accordance with the tenets of the Declaration of Helsinki. This study was registered at Chinese Clinical Trial Registry (registration number: ChiCTR-ROC-17011707).

### 2.2. Questionnaire

All patients were required to fill out a questionnaire (standard patient evaluation of eye dryness (SPEED)) [27] for assessing ocular surface symptoms prior to routine ophthalmic examinations. The scores on the questionnaire ranged from 0 to 28 according to the severity of patients' symptoms. The symptoms included dryness, grittiness or scratchiness, soreness or irritation, burning or watering, and eye fatigue. The frequency of the symptoms was graded as never (0), sometimes (1), often (2), and constantly (3). The subjective sensation of the symptoms was categorized as no problems (0), tolerable (1), uncomfortable (2), bothersome (3), and intolerable (4).

### 2.3. Lipid Layer Thickness and Blink Assessment

The LipiView interferometer (TearScience Inc., Morrisville, NC) was used to capture a 20 s video of interference pattern of the subject's tear film. In addition to counting the subject's total and partial blinks, the interferometer converts the specific interference colors into the values of lipid layer thickness (LLT) [28, 29].

### 2.4. Tear Meniscus Height, Noninvasive Tear Break-Up Time, and Meibography

The Keratograph 5M (Oculus GmbH, Wetzlar, Germany) equipped with a modified tear film-scanning function was used to measure tear meniscus height (TMH) by capturing the lower tear film meniscus images and detect noninvasive tear film break-up time (NI-BUT) as described previously [30]. Furthermore, the subject's upper and lower eyelids were everted, and the high-contrast image of meibomian glands (MGs) was acquired under an infrared meibography model [31]. The MG dropout area was quantified by a meiboscore (grade 0, no dropout; grade 1, <33% dropout; grade 2, 33 to 67% dropout; and grade 3, >67% dropout), and the scores from upper and lower eyelids were added (total meiboscore, range 0∼6) to reflect MG dropout of the eye [32].

### 2.5. Superficial Punctate Keratopathy Score

The severity of corneal surface damage was evaluated by staining the cornea with fluorescein; both the staining area and staining density were scored from 0 to 3 as described previously [4, 33]. The specific criteria are listed in Table 1. The product of both scores was calculated, termed superficial punctuate keratopathy (SPK), and used as an index for the damage of corneal surface.

### 2.6. Corneal Sensitivity

A Cochet–Bonnet aesthesiometer was used to examine corneal sensitivity as described elsewhere [34]. The tip of a fully extended nylon filament was applied to the central cornea at a perpendicular angle, and the thread length was recorded when the subject felt its presence.

### 2.7. Schirmer I Test

Total tear secretion was measured without anesthesia by placing a standardized Schirmer strip into accus conjunctivae at lateral 1/3 of lower lid for 5 min with eyes closed gently, and then the length of the wet strip was recorded.

### 2.8. Statistical Analysis

Statistical analyses were performed using Statistical Program for Social Sciences 20.0 (IBM SPSS Inc., New York, NY, USA). All data were expressed as mean ± SEM. The data were examined using the D'Agostino and Pearson omnibus normality test. The data with a Gaussian distribution were further examined by the Levene test to confirm homogeneity of variance. The differences among the diabetic duration subgroups and the healthy controls were analyzed by one-way ANOVA followed by a Tukey post hoc. For the data with nonparametric distribution, the differences among groups were analyzed by the Kruskal–Wallis test followed by Dunn's post hoc. The associations between the parameters were analyzed by Spearman's correlation analysis. A *P* value less than 0.05 was considered statistically significant.

## 3. Results

### 3.1. General Condition

The gender (*χ*^2^ = 1.015, *P*=0.314) and age (*t* = −1.428, *P*=0.154) did not differ significantly between the diabetes and control groups (Table 2). Moreover, no significant difference was found in gender (*χ*^2^ = 3.854, *P*=0.278), age (*F* = 0.881, *P*=0.452) or percentage of HbA1c (*F* = 1.158, *P*=0.316) among the patients subgrouped according to the diabetic duration and the control group (Table 2). The prevalence of DR in total diabetic patients was 70.9%. The DR incidence was 25% in the patients with diabetes less than 10 y, 85.9% in those with diabetes 10–20 y, and 100% in those with the disease for more than 21 y, suggesting a significantly increased DR incidence as the diabetic duration prolongs (*χ*^2^ = 87.084, *P* ≤ 0.001; Table 2).

### 3.2. Comparison of Ocular Surface Parameters between Diabetics and Healthy Controls

**Figure 1:**
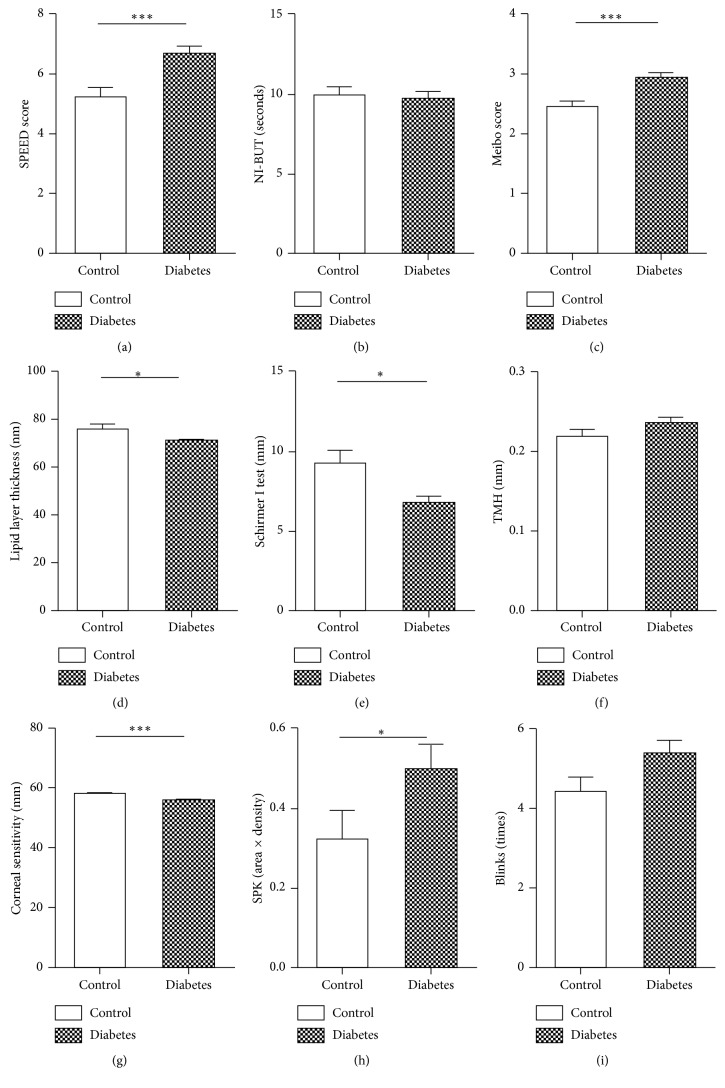
Comparison of ocular surface parameters between diabetic patients and healthy controls. The SPEED score (a), meiboscore (c), lipid layer thickness values (d), Schirmer I results (e), corneal sensitivity (g), and SPK score (h) were significantly different between the diabetic and the healthy control group. The differences in NI-BUT (b), TMH (f), and blinks (i) did not reach statistical significance between the diabetic and control groups (^*∗*^*P* < 0.05, ^*∗∗*^*P* < 0.01, and ^*∗∗∗*^*P* ≤ 0.001).

There were no significant differences in blink frequency, NI-BUT, and TMH values between the diabetes and control groups (Table 3, Figure 1). The SPEED score (*Z* = −3.600, *P* ≤ 0.001) (Figure 1(a)), meiboscore (*t* = −4.003, *P* ≤ 0.001) (Figure 1(c)), and SPK score (*Z* = −2.463, *P*=0.014) (Figure 1(h)) in the diabetic group were significantly higher than those in the control group. In addition, LLT values (*t* = −2.018, *P*=0.045) (Figure 1(d)), Schirmer I test results (*Z* = −1.991, *P*=0.046) (Figure 1(e)), and corneal sensitivity (*t* = −4.100, *P* ≤ 0.001) (Figure 1(g)) were significantly decreased in the diabetic group as compared to the control group.

### 3.3. Comparison for Ocular Surface Parameters among the Diabetic Duration Subgroups and Control Group

**Figure 2:**
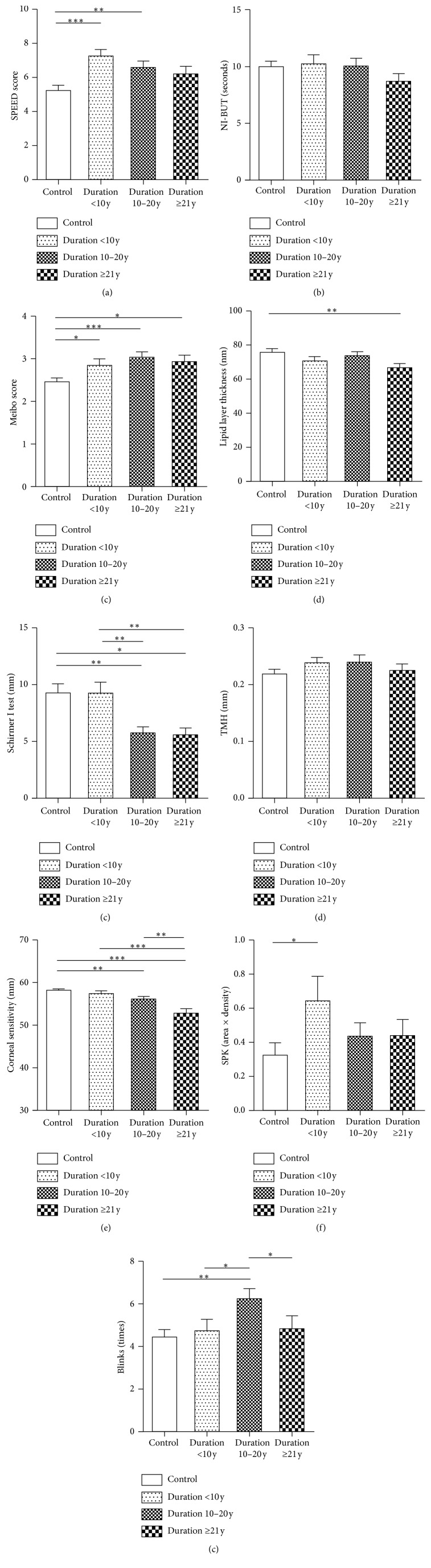
Comparisons of ocular surface parameters among the diabetic duration subgroups and the healthy control group. The SPEED score (a), NI-BUT (b), meiboscore (c), lipid layer thickness (d), Schirmer I test result (e), TMH (f), corneal sensitivity (g), SPK score (h), and blinks (i) were compared among the diabetic subgroups with different disease duration and the healthy control group (^*∗*^*P* < 0.05, ^*∗∗*^*P* < 0.01, and ^*∗∗∗*^*P* ≤ 0.001).

The SPEED score (*H* = 16.630, *P*=0.001), Schirmer I test result (*H* = 14.164, *P*=0.003), corneal sensitivity (*F* = 11.344, *P* ≤ 0.001), meiboscore (*F* = 4.950, *P*=0.002), and blink rate (*H* = 10.232, *P*=0.017) were significantly different among the diabetic subgroups and the healthy control group (Table 3, Figure 2). Other parameters, such as SPK score, NI-BUT, TMH, and LLT values, did not exhibit significant differences among these groups (Table 3, Figure 2). The SPEED score (Figure 2(a)) in the control group was significantly lower than those in the subgroups with diabetic duration <10 y (*Z* = −3.912, *P* ≤ 0.001) and 10 to 20 y (*Z* = −2.510, *P*=0.012). The meiboscore was significantly higher in the subgroups with diabetic duration <10 y (*t* = −2.166, *P*=0.033), duration 10 to 20 y (*t* = −3.675, *P* ≤ 0.001), and duration ≥21 y (*t* = −2.481, *P*=0.015) than that in the healthy controls (Figure 2(c)). The LLT value in the diabetic subgroup with duration ≥21 y was significantly reduced as compared with the controls (*t* = −2.949, *P*=0.004, Figure 2(d)). The value of the Schirmer I test in the control group was significantly higher than those in the subgroups with diabetic duration 10 to 20 y (*Z* = −2.773, *P*=0.006) and duration ≥21 y (*Z* = −2.053, *P*=0.040, Figure 2(e)). Also, the subgroup with diabetic duration <10 y showed a higher Schirmer I test value than those in the subgroups with duration 10 to 20 y (*Z* = −3.000, *P*=0.003) and duration ≥21 y (*Z* = −2.674, *P*=0.007, Figure 2(e)). As for corneal sensitivity, the recorded length of nylon filament in the control group was significantly longer, indicative of higher corneal sensitivity, than those in the subgroups with duration 10 to 20 y (*t* = 2.716, *P*=0.007) and ≥21 y (*t* = 4.640, *P* ≤ 0.001). Moreover, corneal sensitivity in the subgroup with duration ≥21 y was significantly deteriorated as compared to the subgroups with duration <10 y (*t* = 3.605, *P*=0.001) and duration 10 to 20 y (*t* = 2.640, *P*=0.010) (Figure 2(g)). The SPK score was significantly increased in the subgroup with duration <10 y as compared with healthy controls (*Z* = −2.463, *P*=0.014), indicating the diabetes-induced damage on ocular surface (Figure 2(h)). The diabetic subgroup with duration of 10 to 20 y had greater blink frequency than the healthy control group (*Z* = −3.044, *P*=0.002), the diabetic subgroups with the duration <10 y (*Z* = −2.127, *P*=0.033), and duration ≥21 y (*Z* = −2.203, *P*=0.027) (Figure 2(i)).

### 3.4. Correlations of the Ocular Surface Parameters in Diabetic Groups

**Figure 3:**
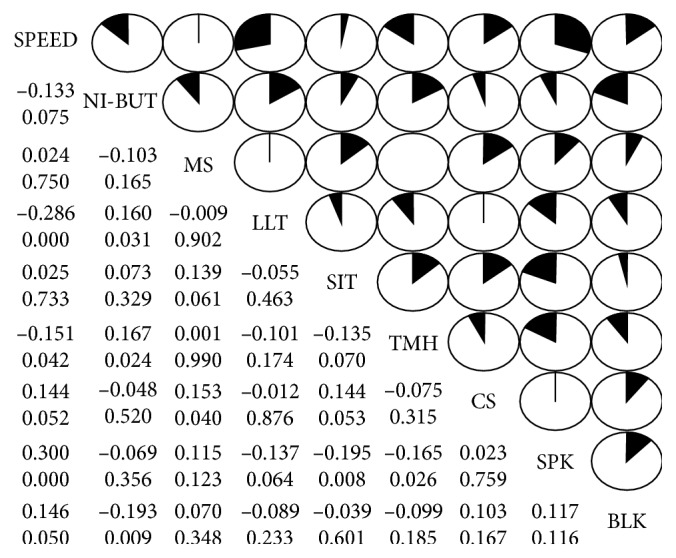
A correlogram illustrates the strength of correlations between ocular surface clinical parameters in the diabetic patients. In the pie graphs, the clockwise direction indicates a positive correlation and counterclockwise direction a negative correlation. For the numbers on the left of the pie graph, the upper one indicates the correlation coefficient and the lower one the *P* value (SPEED, standard patient evaluation of eye dryness; LLT, lipid layer thickness; NI-BUT, noninvasive tear film break-up time; TMH, tear meniscus height; MS, total percentage of meibomian gland dropout area in upper and lowers eyelids; SPK, superficial punctate keratopathy score; CS, corneal sensitivity; SIT, Schirmer I Test (total tear secretion)).

In the diabetic patients, the score of SPEED was positively correlated with the SPK score (*r* = 0.300, *P* ≤ 0.001) and blink rate (*r* = 0.146, *P*=0.050) and negatively correlated with TMH (*r* = −0.151, *P*=0.042) and LLT values (*r* = −0.286, *P* ≤ 0.001) (Figure 3). In addition, corneal sensitivity was positively correlated with the meiboscore (*r* = 0.153, *P*=0.040) and barely correlated with the SPEED score (*r* = 0.144, *P*=0.052) (Figure 3). There were also positive correlations between NI-BUT and TMH (*r* = 0.167, *P*=0.024) as well as between NI-BUT and LLT values (*r* = 0.160, *P*=0.031) (Figure 3). On the other hand, negative correlations were found between NI-BUT and blink frequency (*r* = −0.193, *P*=0.009), between TMH and SPK score (*r* = −0.165, *P*=0.026), as well as between the Schirmer I test value and SPK score (*r* = −0.195, *P*=0.008) (Figure 3).

### 3.5. Correlations between Ocular Surface Parameters and Duration of Diabetes

**Figure 4:**
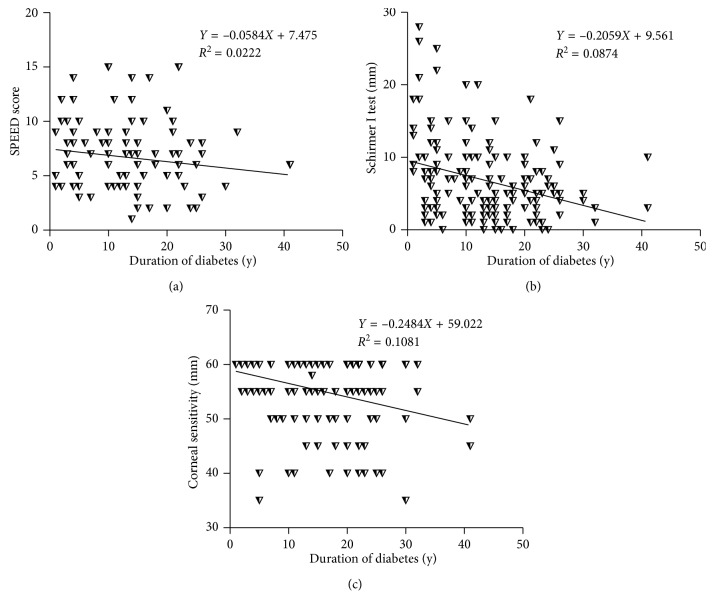
Correlations between ocular surface parameters and duration of diabetes. In the total diabetic group, the SPEED score (a), Schirmer I test (b), and corneal sensitivity (c) were negatively correlated with duration of diabetes.

In the total diabetic group, the Schirmer I test (*r* = −0.268, *P* ≤ 0.001), corneal sensitivity (*r* = −0.336, *P* ≤ 0.001), and SPEED score (*r* = −0.171, *P*=0.021) exhibited negative correlations with duration of diabetes (Figure 4).

## 4. Discussion

Diabetes mellitus is a systemic disease characterized by chronic hyperglycemia and dysregulated metabolism and may lead to LFU dysfunctions through different mechanisms. Patients with diabetes have demonstrated structural, metabolic, and functional abnormalities in the cornea and conjunctiva, which subsequently increase the risk of developing diabetic complications in ocular surface [3–6, 12, 23].

It has been proposed that as the duration of diabetes increases, the risk of developing proliferative diabetic retinopathy [24, 25] and diabetic neuropathy [8–11] increases dramatically; therefore, we would expect the ocular surface parameters measured in this study to become significantly exacerbated as diabetes persists. However, this is not necessarily true based on our results, as varying degrees of deterioration in the LFU, including tear film, ocular surface function, and corneal sensation, were detected in the patients afflicted by diabetes for different periods time (Figure 2, Table 3). The duration of diabetes was only negatively correlated with the SPEED score, Schirmer I test, and corneal sensitivity in the diabetes group (Figure 4, Table 3).

Apart from the diabetic subgroup with the disease duration ≥21 y, we found that the SPEED scores in other diabetes subgroups were significantly greater than the healthy controls (Figure 1(a)). Further, the SPEED score exhibits a trendy correlation with the corneal sensitivity (Figure 3) and a significant negative correlation with the diabetic duration (Figure 4(a)). These results suggest that subjective symptoms became exacerbated in early diabetic patients and then attenuated as the disease persisted; this may be due to the blunted corneal sensitivity caused by peripheral neuropathy during long-term diabetes.

Abnormalities in the quantity and quality in tear secretion have been reported in diabetes, but the results remain controversial. In contrast to the results of other studies, the data in this study showed that the difference in NI-BUT did not reach a statistical significance between diabetes and control group (Figure 1(b)). This was probably because the distinct apparatus and calculation method were used to measure the tear film BUT in the current study; the difference between the diabetics and normal controls might become less dramatic [28]. However, the NI-BUT in this study did exhibit a trendy decrease as diabetes persisted (Figure 2(b), Table 3), which is consistent with the results of previous studies [3, 7, 12–14, 19].

The MGs are large sebaceous glands that are innervated by parasympathetic fibers [35] and produce meibum, a major source of lipid in the tear film [36]. It is generally accepted that diabetic patients are more susceptible to neuropathy [10, 23], as well as blepharitis and recurrent styes resulting from infected sebaceous glands [37, 38]. Indeed, we found, in the current study, that the MG dropout is more severe in all the diabetic groups than the healthy controls (Figure 2(c)) and a positive correlation between MG dropout and corneal sensitivity (Figure 3). Furthermore, the LLT in diabetics was significantly deceased when compared to the controls (Figure 1(d)). Whereas a compromised lipid layer could, in turn, cause excessive evaporation of tear film [39]. Therefore, we speculate that diabetic neuropathy, repeated infection and inflammation, might contribute to obstruction in MG orifices, MG atrophy, and dropout in the patients with a long history of diabetes.

Besides the tear film instability and excessive evaporation, the diminished tear secretion was also observed in diabetic patients [3, 7, 12–14, 19]. Such reduced tear secretion has been considered to be caused by the decreased reflex tear secretion, which is positively correlated to corneal sensation [8, 14]. In our study, the decreased total tear secretion was observed in all diabetes groups, particularly in the subgroups with duration 10 to 20 y and duration ≥21 y when compared to the controls (Figure 2(e)). Moreover, we also found decreased corneal sensitivity in these two diabetic subgroups and a trendy positive correlation between total tear secretion and corneal sensitivity (Figure 3). Furthermore, the total tear secretion is negatively correlated with the duration of diabetes (Figure 4(b)). These results suggest that the reduced total tear secretion may be caused, at least in part, by the impaired reflex tear secretion during long-term diabetes.

Diabetic neuropathy is one of the most common long-term complications of diabetes. As revealed by our result and previous studies [3, 7, 9–12, 19], diabetic patients have decreased corneal sensitivity. Furthermore, our finding revealed the significantly decreased corneal sensitivity in the diabetic subgroups with duration 10 to 20 y and duration ≥21 y as compared with controls (Figure 2(g)), suggesting that the symptoms of diabetic neuropathy often occur after 10 y of the disease onset. In addition, the negative correlation between the duration of diabetes and corneal sensitivity (Figure 4(c)) suggests that long-term hyperglycemia and metabolic syndrome in diabetes may deteriorate corneal sensation. As mentioned above, the deteriorated corneal sensitivity in diabetes leads to declined reflex tear secretion [8]. Moreover, the neurodegeneration in conjunctiva may result in abnormal secretion of mucin proteins from goblet cells, further compromising the quality and stability of tear film [40]. Thirdly, diabetic peripheral neuropathy in ocular surface can cause malnutrition and metabolic abnormalities in cornea, which consequently induces refractory corneal epithelial ulcer and erosion [41]. These factors may form a vicious cycle, exacerbating LFU dysfunction and ocular surface damage as diabetes persists.

Blinking plays an important role in maintaining the stability of tear film and is related to psychological and/or several systemic diseases [4, 20, 42]. In this study, we observed the increased blinking frequencies in all diabetes groups, but only the subgroup with diabetic duration of 10 to 20 y reached statistical significance as compared to the healthy controls (Figure 2(i)). This result was surprising, as one would expect the diabetics to have reduced blinking frequency as a result of the impaired corneal sensation. However, the ocular surface conditions in early diabetes, such as the reduced tear secretion, the unstable tear film caused by disruption in the lipid layer and paucity of mucin proteins, as well as the sterile or nonsterile inflammation, may still boost blinking frequency via the blunted corneal sensation as a compensatory mechanism [42–44]. When diabetes persists for more than 20 y, the corneal sensitivity is so severely damaged by a long-term neuropathy that it cannot elicit blinks in response to the unfavorable conditions, and the blinking frequency hence fell down in these groups of diabetic patients.

In conclusion, our study demonstrates the importance of stratifying diabetic patients based on the disease duration, which has been ignored in the previous studies on ocular surface dysfunctions in the diabetics. In addition, our results show that there are significant differences in multiple ocular parameters among the subgroups with different diabetic durations, thereby indicating that long-term hyperglycemia and dysregulated metabolism may lead to exacerbating tear film abnormalities and ocular surface disorders.

## Figures and Tables

**Table 1:** Classification of severity in corneal epithelial lesions.

Area: corneal surface area	Density: density of damaged lesions
A0: no punctate staining	D0: no punctate staining
A1: less than one-thirds	D1: sparse density
A2: one third to two-thirds	D2: moderate density
A3: more than two-thirds	D3: high density with overlapping lesions

**Table 2:** Demographics of diabetic subgroups and control group.

Demographics	Controls	Diabetic patients			
		<10 y	10∼20 y	≥21 y	Total
Subject (*n*)	51	28	39	24	91
M : F ratio	18 : 33	11 : 17	18 : 21	14 : 10	40 : 51
Age (y)	64.35 ± 5.66	64.93 ± 6.03	65.54 ± 6.41	65.83 ± 6.57	65.43 ± 6.31
HbA1c (%)	—	6.94 ± 0.43	7.00 ± 0.51	6.87 ± 0.53	6.95 ± 0.49
Duration (y)	0	4.18 ± 2.02	13.67 ± 2.46	24.22 ± 4.81	13.58 ± 8.27
DR (no. of eyes)	0	56	78	48	182
NDR (*n* (%))	0	42 (75)	11 (14.1)	0	53 (29.1)
NPDR (*n* (%))	0	14 (25)	67 (85.9)	48 (100)	129 (70.9)
PDR (%)	0	0	0	0	0

Note: duration, duration of diabetes; DR, diabetic retinopathy; NDR, no diabetic retinopathy; NPDR, nonproliferative diabetic retinopathy; PDR, proliferative diabetic retinopathy.

**Table 3:** Ocular surface clinical parameters in diabetic patients and normal controls.

Parameters	Controls	Diabetic patients			
		<10 y	10∼20 y	≥21 y	Total
SPEED	5.24 ± 3.04	7.25 ± 2.99	6.56 ± 3.48	6.21 ± 3.10	6.68 ± 3.24
LLT (nm)	75.96 ± 19.79	70.71 ± 20.15	73.69 ± 22.07	66.71 ± 16.98	71.04 ± 20.46
Blinks (times)	4.43 ± 3.57	4.73 ± 4.05	6.23 ± 4.25	4.81 ± 4.28	5.40 ± 4.24
NI-BUT (sec)	9.97 ± 5.19	10.22 ± 6.13	10.02 ± 6.33	8.68 ± 4.85	9.73 ± 5.91
TMH (mm)	0.22 ± 0.86	0.24 ± 0.75	0.24 ± 0.11	0.23 ± 0.81	0.24 ± 0.10
Meiboscore	2.46 ± 0.86	2.84 ± 1.14	3.03 ± 1.13	2.92 ± 1.13	2.94 ± 1.13
SPK score	0.32 ± 0.73	0.64 ± 1.09	0.44 ± 0.69	0.44 ± 0.68	0.50 ± 0.83
Sensitivity	58.19 ± 4.15	57.41 ± 5.04	56.13 ± 5.62	52.81 ± 7.50	55.65 ± 6.24
Schirmer (mm)	9.25 ± 8.07	9.23 ± 7.18	5.73 ± 4.89	5.56 ± 4.20	6.76 ± 5.76

*Note*. SPEED, standard patient evaluation of eye dryness; LLT, lipid layer thickness; NI-BUT, noninvasive tear film break-up time; TMH, tear meniscus height; meiboscore, total percentage of meibomian gland dropout area in upper and lowers eyelids; SPK score, superficial punctate keratopathy score; sensitivity, corneal sensitivity; Schirmer, Schirmer I test (total tear secretion).

## Data Availability

The data used to support the findings of this study are included within the article.

## References

[1] Stern M. E., Beuerman R. W., Fox R. I., Gao J., Mircheff A. K., Pflugfelder S. C. (1998). The pathology of dry eye: the interaction between the ocular surface and lacrimal glands. *Cornea*.

[2] (2007). Research in dry eye: report of the research subcommittee of the international dry eye WorkShop (2007). *Ocular Surface*.

[3] Yoon K. C., Im S. K., Seo M. S. (2004). Changes of tear film and ocular surface in diabetes mellitus. *Korean Journal of Ophthalmology*.

[4] Inoue K., Okugawa K., Amano S (2005). Blinking and superficial punctate keratopathy in patients with diabetes mellitus. *Eye*.

[5] Nepp J., Abela C., Polzer I., Derbolav A., Wedrich A. (2000). Is there a correlation between the severity of diabetic retinopathy and keratoconjunctivitis sicca?. *Cornea*.

[6] Inoue K., Kato S., Ohara C., Numaga J., Amano S., Oshika T. (2001). Ocular and systemic factors relevant to diabetic keratoepitheliopathy. *Cornea*.

[7] Lv H., Li A., Zhang X. (2014). Meta-analysis and review on the changes of tear function and corneal sensitivity in diabetic patients. *Acta Ophthalmologica*.

[8] Saito J., Enoki M., Hara M., Morishige N., Chikama T., Nishida T. (2003). Correlation of corneal sensation, but not of basal or reflex tear secretion, with the stage of diabetic retinopathy. *Cornea*.

[9] Neira-Zalentein W., Holopainen J. M., Tervo T. M (2011). Corneal sensitivity in diabetic patients subjected to retinal laser photocoagulation. *Investigative Opthalmology & Visual Science*.

[10] Pritchard N., Edwards K., Vagenas D (2010). Corneal sensitivity as an ophthalmic marker of diabetic neuropathy. *Optometry and Vision Science*.

[11] Cousen P., Cackett P., Bennett H., Swa K., Dhillon B. (2007). Tear production and corneal sensitivity in diabetes. *Journal of Diabetes and its Complications*.

[12] Dogru M., Katakami C., Inoue M. (2001). Tear function and ocular surface changes in noninsulin-dependent diabetes mellitus. *Ophthalmology*.

[13] Yu L., Chen X., Qin G., Xie H., Lv P. (2008). Tear film function in type 2 diabetic patients with retinopathy. *Ophthalmologica*.

[14] Goebbels M. (2000). Tear secretion and tear film function in insulin dependent diabetics. *British Journal of Ophthalmology*.

[15] Liu J., Shi B., He S., Yao X., Willcox M. D., Zhao Z. (2010). Changes to tear cytokines of type 2 diabetic patients with or without retinopathy. *Molecular Vision*.

[16] Park K. S., Kim S. S., Kim J. C (2008). Serum and tear levels of nerve growth factor in diabetic retinopathy patients. *American Journal of Ophthalmology*.

[17] Zhao Z., Liu J., Shi B., He S., Yao X., Willcox M. D. (2010). Advanced glycation end product (AGE) modified proteins in tears of diabetic patients. *Molecular Vision*.

[18] Kim H. J., Kim P. K., Yoo H. S., Kim C. W. (2012). Comparison of tear proteins between healthy and early diabetic retinopathy patients. *Clinical Biochemistry*.

[19] Misra S. L., Patel D. V., McGhee C. N. (2014). Peripheral neuropathy and tear film dysfunction in type 1 diabetes mellitus. *Journal of Diabetes Research*.

[20] Eissa I. M., Khalil N. M., El-Gendy H. A. (2016). A controlled study on the correlation between tear film volume and tear film stability in diabetic patients. *Journal of Ophthalmology*.

[21] Manaviat M. R., Rashidi M., Afkhami-Ardekani M., Shoja M. R. (2008). Prevalence of dry eye syndrome and diabetic retinopathy in type 2 diabetic patients. *BMC Ophthalmology*.

[22] Ozdemir M., Buyukbese M. A., Cetinkaya A., Ozdemir G. (2003). Risk factors for ocular surface disorders in patients with diabetes mellitus. *Diabetes Research and Clinical Practice*.

[23] Najafi L., Malek M., Valojerdi A. E (2013). Dry eye and its correlation to diabetes microvascular complications in people with type 2 diabetes mellitus. *Journal of Diabetes and Its Complications*.

[24] Voutilainen-Kaunisto R. M., Terasvirta M. E., Uusitupa M. I., Niskanen L. K. (2001). Occurrence and predictors of retinopathy and visual acuity in Type 2 diabetic patients and control subjects. *Journal of Diabetes and Its Complications*.

[25] Rani P. K., Raman R., Chandrakantan A., Pal S. S., Perumal G. M., Sharma T. (2009). Risk factors for diabetic retinopathy in self-reported rural population with diabetes. *Journal of Postgraduate Medicine*.

[26] (1991). Grading diabetic retinopathy from stereoscopic color fundus photographs—an extension of the modified Airlie House classification. ETDRS report number 10. Early Treatment Diabetic Retinopathy Study Research Group. *Ophthalmology*.

[27] Ngo W., Situ P., Keir N., Korb D., Blackie C., Simpson T. (2013). Psychometric properties and validation of the standard patient evaluation of eye dryness questionnaire. *Cornea*.

[28] Finis D., Pischel N., Schrader S., Geerling G. (2013). Evaluation of lipid layer thickness measurement of the tear film as a diagnostic tool for Meibomian gland dysfunction. *Cornea*.

[29] Blackie C. A., Solomon J. D., Scaffidi R. C., Greiner J. V., Lemp M. A., Korb D. R. (2009). The relationship between dry eye symptoms and lipid layer thickness. *Cornea*.

[30] Hong J., Sun X., Wei A (2013). Assessment of tear film stability in dry eye with a newly developed keratograph. *Cornea*.

[31] Menzies K. L., Srinivasan S., Prokopich C. L., Jones L. (2015). Infrared imaging of meibomian glands and evaluation of the lipid layer in Sjogren’s syndrome patients and nondry eye controls. *Investigative Ophthalmology & Visual Science*.

[32] Korb D. R., Blackie C. A. (2013). Debridement-scaling: a new procedure that increases Meibomian gland function and reduces dry eye symptoms. *Cornea*.

[33] Miyata K., Amano S., Sawa M., Nishida T. (2003). A novel grading method for superficial punctate keratopathy magnitude and its correlation with corneal epithelial permeability. *Archives of Ophthalmology*.

[34] Golebiowski B., Papas E., Stapleton F. (2011). Assessing the sensory function of the ocular surface: implications of use of a non-contact air jet aesthesiometer versus the Cochet-Bonnet aesthesiometer. *Experimental Eye Research*.

[35] Chung C. W., Tigges M., Stone R. A. (1996). Peptidergic innervation of the primate meibomian gland. *Investigative Ophthalmology and Visual Science*.

[36] Geerling G., Tauber J., Baudouin C (2011). The international workshop on meibomian gland dysfunction: report of the subcommittee on management and treatment of meibomian gland dysfunction. *Investigative Opthalmology and Visual Science*.

[37] Clifford C. W., Fulk G. W. (1990). Association of diabetes, lash loss, and *Staphylococcus aureus* with infestation of eyelids by *Demodex folliculorum* (Acari: demodicidae). *Journal of Medical Entomology*.

[38] Skarbez K., Priestley Y., Hoepf M., Koevary S. B. (2010). Comprehensive review of the effects of diabetes on ocular health. *Expert Review of Ophthalmology*.

[39] Craig J. P., Tomlinson A. (1997). Importance of the lipid layer in human tear film stability and evaporation. *Optometry and Vision Science*.

[40] Alessandro L., Alessandra M., Graziella P. (2009). In vitro evidence of nerve growth factor effects on human conjunctival epithelial cell differentiation and mucin gene expression. *Investigative Opthalmology and Visual Science*.

[41] Nishida T., Chikama T., Sawa M. (2012). Differential contributions of impaired corneal sensitivity and reduced tear secretion to corneal epithelial disorders. *Japanese Journal of Ophthalmology*.

[42] Rahman E. Z., Lam P. K., Chu C. K., Moore Q., Pflugfelder S. C. (2015). Corneal sensitivity in tear dysfunction and its correlation with clinical parameters and blink rate. *American Journal of Ophthalmology*.

[43] Nakamori K., Odawara M., Nakajima T., Mizutani T., Tsubota K. (1997). Blinking is controlled primarily by ocular surface conditions. *American Journal of Ophthalmology*.

[44] Al-Abdulmunem M. (1999). Relation between tear breakup time and spontaneous blink rate. *International Contact Lens Clinic*.

